# Isolation of Complete Equine Encephalitis Virus Genome from Human Swab Specimen, Peru

**DOI:** 10.3201/eid2408.171274

**Published:** 2018-08

**Authors:** Diana Juarez, Carolina Guevara, Michael Wiley, Armando Torre, Gustavo Palacios, Eric S. Halsey, Sonia Ampuero, Mariana Leguia

**Affiliations:** United States Naval Medical Research Unit No. 6, Lima, Peru (D. Juarez, C. Guevara, A. Torre, E.S. Halsey, S. Ampuero, M. Leguia);; Laboratorio de Genómica, Pontificia Universidad Católica del Perú, Lima (D. Juarez, A. Torre, M. Leguia);; United States Army Medical Research Institute for Infectious Diseases, Frederick, Maryland, USA (M. Wiley, G. Palacios)

**Keywords:** Venezuelan equine encephalitis virus, VEEV, complete genome, next-generation sequencing, pathogen discovery, viruses, vector-borne infections, Peru

## Abstract

While studying respiratory infections in Peru, we identified Venezuelan equine encephalitis virus (VEEV) in a nasopharyngeal swab, indicating that this alphavirus can be present in human respiratory secretions. Because VEEV may be infectious when aerosolized, our finding is relevant for the management of VEEV-infected patients and for VEEV transmission studies.

Venezuelan equine encephalitis virus (VEEV) is one of many alphaviruses transmitted through the bite of infected mosquitoes (1,2). VEEV primarily infects equine species, causing severe encephalitis and death. VEEV may also infect humans, causing fever and influenza-like symptoms that include headache, chills, myalgia, nausea, and vomiting. In severe cases, human VEEV infection may result in neurologic complications that lead to fatalities. Acute VEEV infection is usually confirmed by PCR, sequencing from blood (*3*), or both, or in the case of encephalitis, from spinal fluid. Nasopharyngeal swabs are rarely tested for alphaviruses like VEEV because they are considered nontraditional sample types for these kinds of pathogens.

The US Naval Medical Research Unit No. 6, in coordination with the local ministry of Hhealth, conducts routine surveillance for respiratory and febrile pathogens under Institutional Review Board–approved protocols that comply with all applicable federal regulations governing the protection of human subjects. As part of these efforts, nasopharyngeal swabs and serum samples are tested for a variety of possible etiologies. Frequently, however, a particular sample fails to yield a recognized etiology. This result happens in several scenarios: a patient is infected with an unknown pathogen or with a pathogen for which there is no known diagnostic; a known pathogen has mutated and changed such that a known diagnostic is no longer effective; or a pathogen is present at a concentration too low to diagnose. In these cases, it may be possible to identify the potential etiology using approaches that can identify novel or divergent pathogens, like unbiased next-generation sequencing (NGS).

In 2013, in Iquitos, Peru, we identified a 16-year-old boy who reported a variety of undifferentiated illness symptoms, including fever, chills, general malaise, myalgia, headache, rhinorrhea, sore throat, nausea, vomiting, abdominal pain, retroorbital pain, rash, and photophobia. A field-administered rapid test for influenza on a nasopharyngeal swab specimen was negative. More thorough laboratory tests, including a highly multiplexed MassTag PCR assay that can detect >20 viral and bacterial pathogens (*4*), were also negative (Figure, panel A). The sample entered a pathogen discovery pipeline routinely used to amplify and identify unknown etiologies (*5*). In brief, we cultured 200 μL of the original nasopharyngeal sample in Vero-E6, LLCMK2, and MDCK cells. Cytopathic effect appeared by day 4 in Vero-E6 and LLCKM2 cells and by day 19 in MDCK cells. We used culture supernatant from day 4 LLCMK2 cells as starting material for NGS MiSeq libraries, which we prepared, sequenced, and processed as described (*5*). Sequencing generated 704,444 raw reads, from which 28,555 were de novo assembled into a single 11,412-nt contig (Figure, panel B). When blasted, the contig matched the complete genome of a VEEV-ID strain isolated in Peru from 1994 (GenBank accession no. KC344526.1) with 99% identity. 

**Figure:**
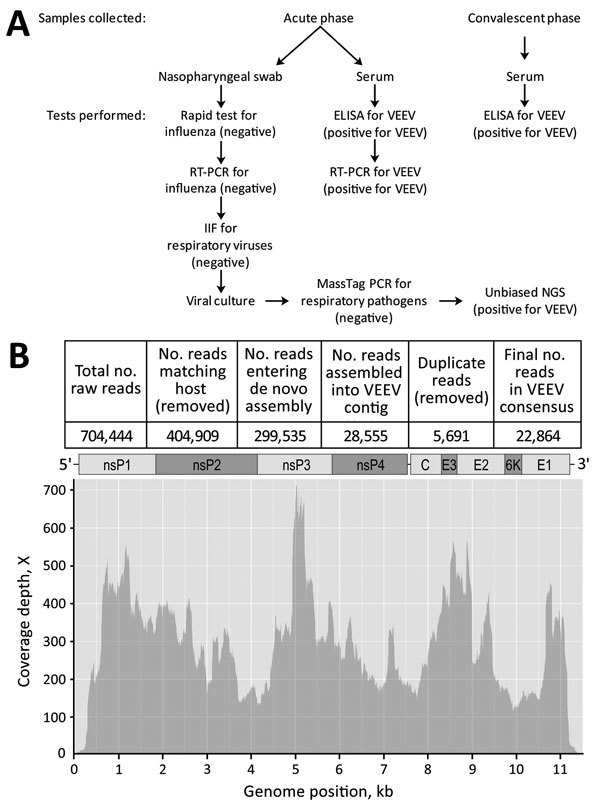
Testing for respiratory pathogens in a 16-year-old boy in Iquitos, Peru. A) Testing algorithm showing the types of samples collected during both acute- and convalescent-phase periods, the tests performed on each, and the results (in parentheses). B) Depth of coverage plot and schematic representation of the isolated VEEV genome, including all genes (nsP1, nsP2, nsP3, nsP4, C, E3, E2, 6K, and E1), drawn to approximate scale. Genomic information for this isolate has been deposited in GenBank (accession no. MF590066). Flu, influenza virus; IIF, indirect immunofluorescence; NGS, next-generation sequencing; VEEV, Venezuelan equine encephalitis virus.

Given the health implications of detecting live alphaviruses in human respiratory secretions, we sought to confirm the finding by retesting the original nasopharyngeal swab, and by examining available paired serum samples collected during acute and convalescent periods. All confirmatory tests we conducted (*3*) were positive for VEEV: the original swab and the acute serum sample tested positive for VEEV by reverse transcription PCR and confirmatory Sanger sequencing; the acute-phase serum sample enabled isolation of VEEV; and the convalescent-phase serum sample showed a 1:1,600 rise in IgM titer, indicating the patient had seroconverted. Subsequent inspection of the associated metadata also indicated the sample had been collected at a time when VEEV circulated in the region.

Febrile surveillance studies have shown that VEEV is prevalent in many countries throughout Latin America (*3*,*6*). Although respiratory symptoms have been reported in association with VEEV infection (*7*) and there is >1 report of VEEV in throat swabs (*8*), human respiratory secretions are seldom tested for alphaviruses. In fact, the presence of VEEV in respiratory secretions, as well as its implications for health and biosafety, are rarely discussed. Although person-to-person transmission of VEEV has not been documented, VEEV can be infectious through aerosolized particles (*9*,*10*), so the potential for an alternate transmission route exists. This possibility should be considered both during individual management of VEEV-infected patients and in studies considering VEEV transmission dynamics or prevention strategies.
